# Nicoliella lavandulae sp. nov., a novel fructophilic Nicoliella species isolated from flowers of Lavandula angustifolia

**DOI:** 10.1099/ijsem.0.006497

**Published:** 2024-08-27

**Authors:** Cristina Alcántara, Ángela Peirotén, Luis A. Ramón-Nuñez, José M. Landete, Manuel Zúñiga, Vicente Monedero

**Affiliations:** 1 Laboratorio de Bacterias Lácticas y Probióticos, Instituto de Agroquímica y Tecnología de Alimentos (IATA-CSIC), Av. Agustín Escardino 7, 46980 Paterna, Spain; 2 Departamento de Tecnología de Alimentos, National Institute for Agricultural and Food Research and Technology (INIA-CSIC), Carretera de La Coruña Km 7.5, 28040 Madrid, Spain; 3 Valencian Institute for Agricultural Research (IVIA), 46113 Valencia, Spain

**Keywords:** flower, fructophilic, *Lavandula angustifolia*, *Nicoliella*, Valencia

## Abstract

A survey of fructophilic lactic acid bacteria associated with wild and cultivated plants in the metropolitan area of Valencia (Spain) led to the isolation of a novel strain of the genus *Nicoliella*, named Es01^T^, from flowers of *Lavandula angustifolia*. The genus *Nicoliella* encompasses a single species, *Nicoliella spurrieriana*. Partial 16S rRNA coding gene sequencing revealed a similarity of 98.8% to *N. spurrieriana* SGEP1_A5^T^. Average nucleotide identity (ANI) calculations revealed an ANI value of 80.49% with strain SGEP1_A5^T^, the only *N. spurrieriana* strain with an available genomic sequence. A digital DNA–DNA hybridization value of 20% was estimated by the Type Strain Genome Server tool when Es01^T^ was compared with strain SGEP1_A5^T^. On the basis of these results, strain Es01^T^ represents a novel species, for which the name *Nicoliella lavandulae* sp. nov. is proposed with Es01^T^ (=CECT 30999^T^=DSMZ 117325^T^=CCM 9394^T^) as type strain.

## Introduction

Fructophilic lactic acid bacteria (FLAB) constitute a heterogeneous group within family *Lactobacillaceae* with unique characteristics, namely, poor growth on d-glucose unless an electron acceptor is available (pyruvate or oxygen, for example) and a preference for d-fructose as a substrate [1,3]. This is due to their obligately heterofermentative metabolism of sugars and lack of a functional alcohol dehydrogenase (AdhE), which is required to maintain the balance between NAD^+^ and NADH in the heterofermentative pathway [4,5]. Heterofermentative lactic acid bacteria (LAB) use the phosphoketolase pathway for sugar metabolism. Phosphoketolase cleaves xylulose-5-phosphate into glyceraldehyde-3-phosphate and acetyl phosphate, which can be converted to ethanol via acetyl-CoA and acetaldehyde. This branch of the heterofermentative pathway allows the reoxidation of the excess of NADH produced in the first steps of the pathway [6]. For this reason, FLAB cannot grow on glucose unless a suitable substrate for NAD regeneration is available. On the other hand, FLAB and most heterofermentative LAB can reduce d-fructose to mannitol by the activity of mannitol dehydrogenase (Mdh), thereby reoxidizing the excess of NADH produced during sugar degradation via the phosphoketolase pathway [3,6]. FLAB encompass all species belonging to the genus *Fructobacillus* and some species of the genus *Apilactobacillus* [3]. In addition, *Nicoliella spurrieriana* has also been described as an obligately fructophilic species [7]. FLAB can be isolated from fructose-rich niches such as flowers, fruits and fermented fruit products [2]. Interestingly, they have also been isolated from the gastrointestinal tract of insects feeding on nectar or fruits, indicating their ability to colonize this niche [3].

A survey of the population of FLAB (September 2022) associated with flowering plants in the metropolitan area of Valencia (39° 40′ 22.7″ N 0° 36′ 42.1″ W), Spain, led to the isolation of strain Es01^T^. Preliminary identification indicated that it belonged to genus *Nicoliella* but, displayed distinctive characteristics that set it apart of *N. spurrieriana*, the only species of the genus described so far. *N. spurrieriana* was originally named *Nicolia spurrieriana* [7]. Subsequently, the genus name was changed to *Nicoliella* to avoid homonymy with previously named genera [8]. *N. spurrieriana* SGEP1_A5^T^ was isolated from the honey of the stingless bee *Tetragonula carbonaria* [7].

## Strain isolation

Strain Es01^T^ was isolated from flowers of *Lavandula angustifolia* (narrow-leaved lavender). Flowers were aseptically collected and placed in tubes containing 5 ml modified FYP broth (10 g d-fructose, 10 g yeast extract, 2.5 g tryptone, 2.5 g meat extract, 2 g sodium acetate, 0.5 g Tween 80, 0.2 g MgSO_4_·7H_2_O, 0.01 g MnSO_4_·4H_2_O, 0.01 g FeSO_4_·7H_2_O, 0.01 g NaCl, 0.05 g cycloheximide and 0.05 g sodium azide per litre; pH 6.8) [1]. The tubes were stirred vigorously and incubated at 30 °C until appreciable growth was observed. Aliquots were conveniently diluted in NaCl 0.9% and spread onto FYP agar plates supplemented with 5 g l^−1^ CaCO_3_, which were subsequently incubated at 30 °C. Isolated colonies were picked on the basis of morphological characteristics and the presence of clearance halos, indicative of acid production. The selected colonies were streaked on FYP agar plates to ensure culture homogeneity, and a single colony was inoculated in FYP broth and incubated at 30 °C for 24 h. Grown cultures were supplemented with glycerol (15% v/v final concentration) and stored at −80 °C.

**Fig. 1.**
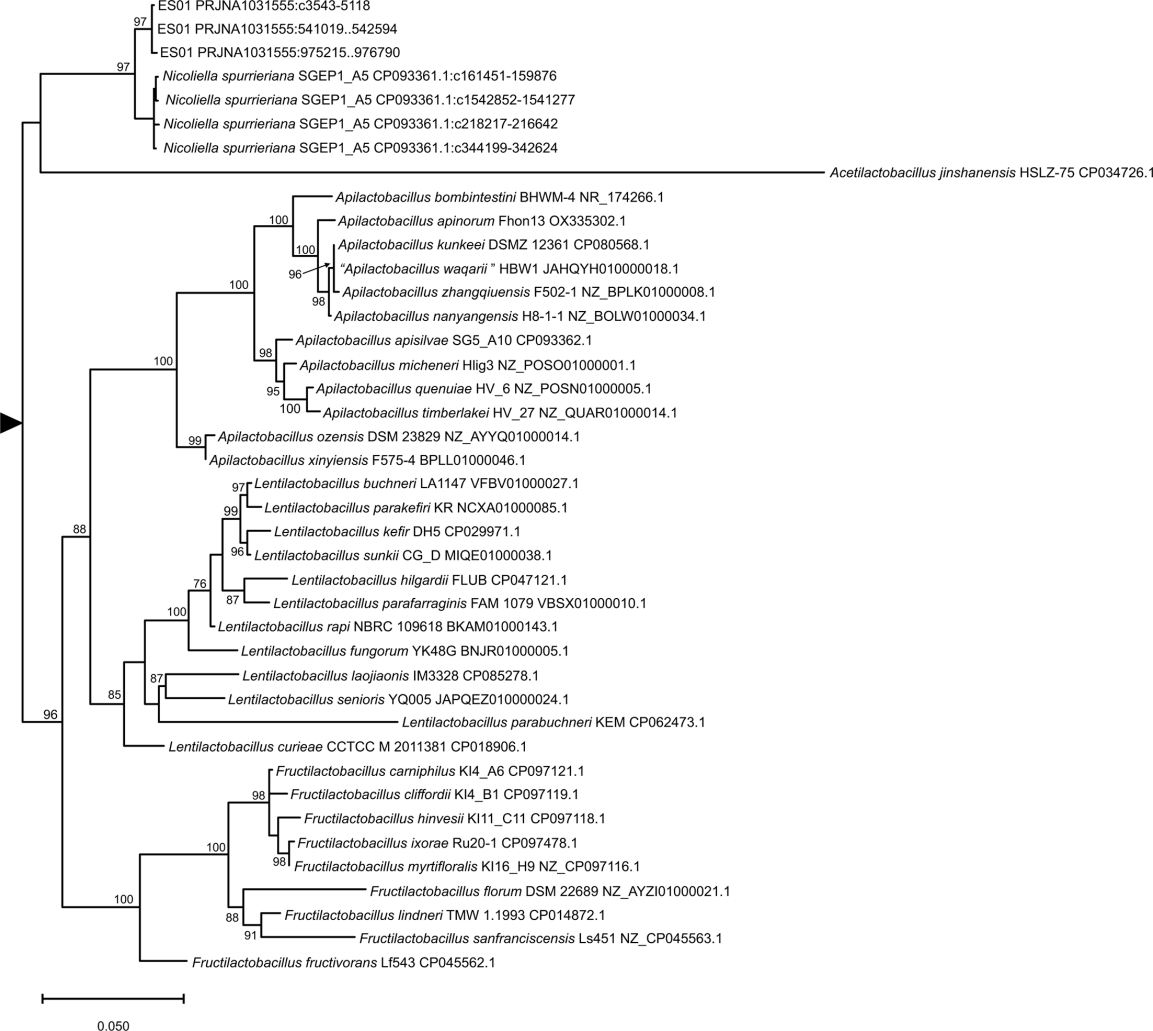
16S rRNA tree of *Nicoliella* and strains of closely related genera. Support values are given for those nodes with support higher than 75%. The tree has been arbitrarily rooted for ease of visualization.

For preliminary identification of isolates, 16S rRNA genes were amplified by PCR with primers 27F (5′-AGAGTTTGATCCTGGCTCAG-3′) and 1492R (5′-GGTTACCTTGTTACG ACTT-3′) [9]. The resulting fragments were sequenced and compared with 16S rRNA gene sequences available in the GenBank databases using the blast sequence alignment tool. Based on the blast result, Es01^T^ was provisionally assigned to the genus *Nicoliella*. The full-length 16S rRNA sequences of Es01^T^ were subsequently extracted from the whole genome sequence (see below) and used to obtain a 16S rRNA based phylogeny by using the maximum-likelihood method. Es01^T^ encodes five copies of the 16S rRNA gene and sequence heterogeneity was detected. Sequence heterogeneity was also observed in the five copies of the 16S rRNA gene of * N. spurrieriana*. Pairwise blast alignments between Es01^T^ and *N. spurrieriana* 16S rRNA sequences were carried out with the global align tool of the NCBI blast server and revealed identities ranging from 98.86% (1558 identical bases out of 1576) to 98.6%(1554 out of 1576) (Table S1, available in the online Supplementary Material). Representative sequences of each variant found in Es01^T^ and *N. spurrieriana* were included in the phylogenetic analysis. Representative species of the most closely related genera, according to the phylogenetic reconstruction of family *Lactobacillaceae* performed by Zheng *et al.* [10], were also selected. All sequences were extracted from GenBank. Alignment using muscle and selection of the best substitution model using the maximum-likelihood method were carried out in mega 11.0.3 [11]. The maximum-likelihood tree was reconstructed by iq-tree [12] with the Kimura two-parameter model [13] using empirical base frequencies and a discrete gamma distribution. Support values were obtained by the ultrafast bootstrap approximations [14]. The phylogenetic analysis confirmed that strain Es01^T^ belongs to the genus *Nicoliella*. The 16S rRNA sequences from Es01^T^ clustered together and apart from their *N. spurrieriana* counterparts (Fig. 1), supporting the status of Es01^T^ as representing a different species.

## Genome features of strain Es01^T^

Total DNA of strain Es01^T^ was extracted using an in-house cetyltrimethylammonium bromide based purification method (to be published elsewhere) and quantified using a Qubit version 3 fluorometer (Invitrogen Life Technologies). Sequencing libraries were prepared using Ligation Sequencing Kit version 14 (SQK-LSK114, Oxford Nanopore) with barcoding SQK-NBD114.24. The generated libraries were sequenced using MinION equipment with an FLO-MIN114 R10.4.1 Flow Cell (Oxford Nanopore) and duplex base-calling in super-accuracy mode selected. The mean read size was 4438.3 bp with a mean read quality of 21.5, as assessed with Nanoplot version 1.36.2 [15]. Reads were assembled with Flye version 2.9.1 [16]. The assembly was deposited in DDBJ/ENA/GenBank (BioProject PRJNA1031555) and annotated by the NCBI Prokaryotic Genome Annotation Pipeline [17].

A total of 1 881 854 pb were sequenced and assembled in four contigs (contig N50, 1.4 Mb; contig L50, 1; genome coverage, 34.0×) with a G+C content of 38.64 mol%. Completeness and contamination were assessed with CheckM [18] and reported as 97.49 and 0.65 %, respectively. Completeness was also estimated with busco [19] as implemented in the Galaxy platform [20]. A completeness of 98.26% was estimated. These values are within the thresholds (≥95% complete with ≤5% contamination) considered acceptable for taxonomy assignments [18]. Our data meet the criteria for use of genomic data for the taxonomy of prokaryotes [21,22]. The genome of *N. spurrieriana* SGEP1_A5^T^ is the only genome sequence available for this species and comprises 2 053 587 bp with 42.11 mol% G+C content. The genome of *N. spurrieriana* (GenBank assembly GCA_023380205.1) is organized in a chromosome of 1 709 727 bp and a secondary chromosome or megaplasmid of 3 438 60 bp. Although the Es01^T^ genome sequence is not complete, the assembly produced two large contigs of 1 418 589 and 4 122 59 bp, suggesting a genetic organization similar to *N. spurrieriana*. Analysis of the Es01^T^ sequence identified 1692 protein encoding genes, 22 pseudogenes and 86 RNA encoding genes, which included five rRNA operons, 68 tRNAs and three ncRNAs. A CRISPR-Cas system of the Type IE class was also present in the Es01^T^ genome at the level of the secondary chromosome (from locus R4146_08045 to R4146_08080), showing a cluster of 29 bp (5′-CTTTTTCTCGCAAGCGCGAGAGTGATCCT-3′) sequence repetitions with 37 spacers. A prophage from the family *Myoviridae* (from locus R4146_07620 to R4146_07805) was also found inserted in the secondary chromosome.

A core genome phylogeny was obtained to more accurately determine the taxonomic status of strain Es01^T^. Reconstruction of the phylogeny was derived by concatenating 20 validated core genes. The validated core gene set selected by Tian and Imanian [23] was used. Protein sequences were extracted, aligned and concatenated by using VBCG version 1.3 [23]. The best substitution model was selected by the maximum-likelihood algorithm in mega 11.0.3. The maximum-likelihood tree was reconstructed with iq-tree with the LG substitution model [24] considering invariants, empirical base frequencies and a discrete gamma distribution. Support values were obtained by ultrafast bootstrap approximation as implemented in iq-tree [12].

**Fig. 2.**
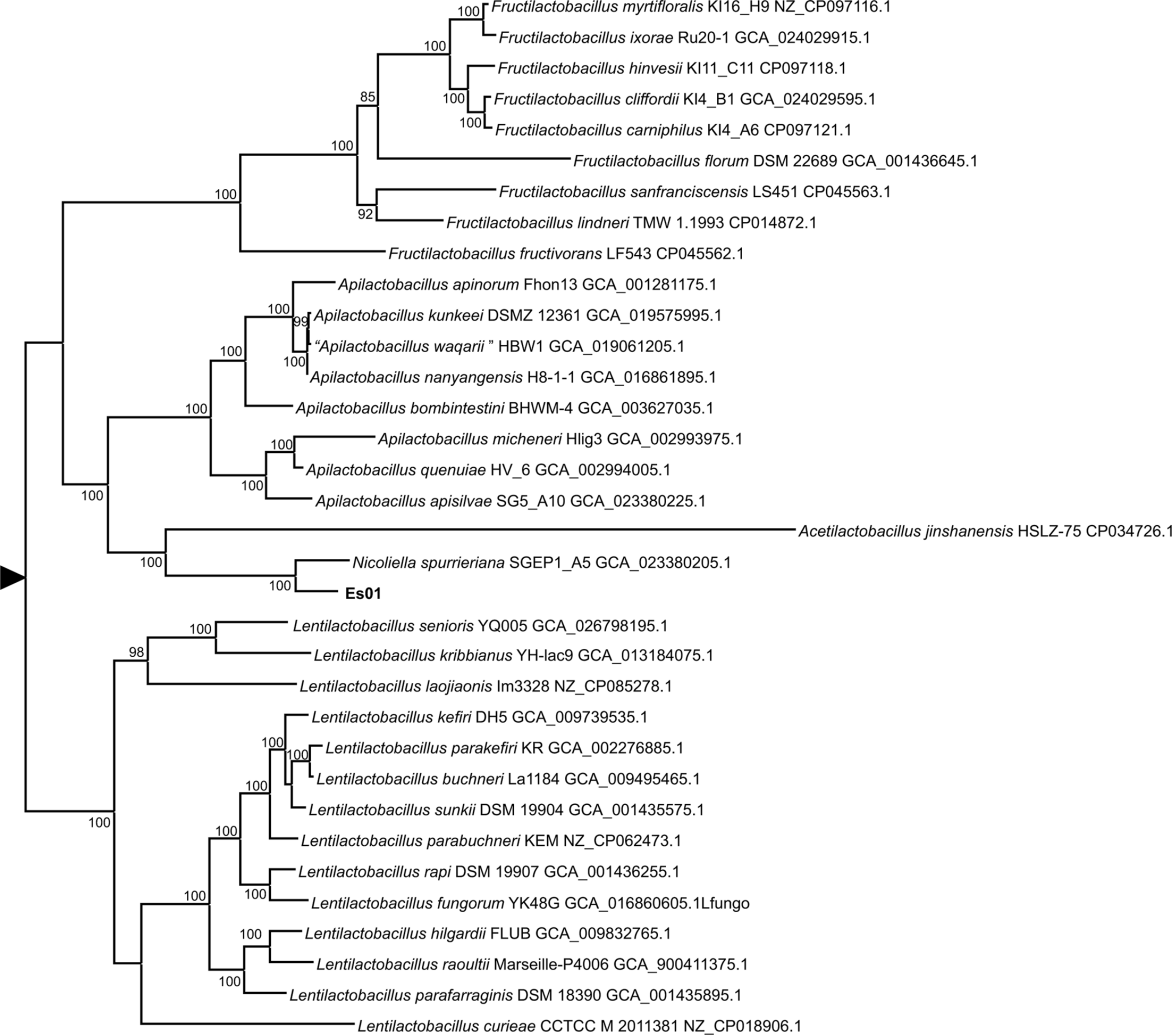
Maximum-likelihood phylogenetic tree of core genes of *Nicoliella* and species of closely related genera. Support values are given for those nodes with support higher than 75%. The tree has been arbitrarily rooted for ease of visualization.

As previously observed in the 16S rRNA gene phylogeny, strain Es01^T^ clustered with *N. spurrieriana* SGEP1_A5^T^ and the phylogenetic distance between the two genomes suggested that Es01^T^ represents a different species (Fig. 2). In order to ascertain the taxonomic status of strain Es01^T^, average nucleotide identity values (ANI) were calculated with FastANI [25], as implemented in Proksee [26], and OrthoANI [27]. The ANI values between strain Es01^T^ and *N. spurrieriana* SGEP1_A5^T^ calculated with FastANI and OrthoANI were 80.49 and 76.20, respectively. These values are below the proposed threshold (95–96 %) for bacterial species [25,28]. In addition, digital DNA–DNA hybridization (dDDH) was carried out with TYGS [29]. When compared with *N. spurrieriana* SGEP1_A5^T^, a value of 20% (CI 17.8–22.4%) was obtained with formula d_4_, which is recommended for incomplete genomes [30]. This value is also below the threshold for bacterial species.

## Growth characteristics of strain Es01^T^

**Fig. 3.**
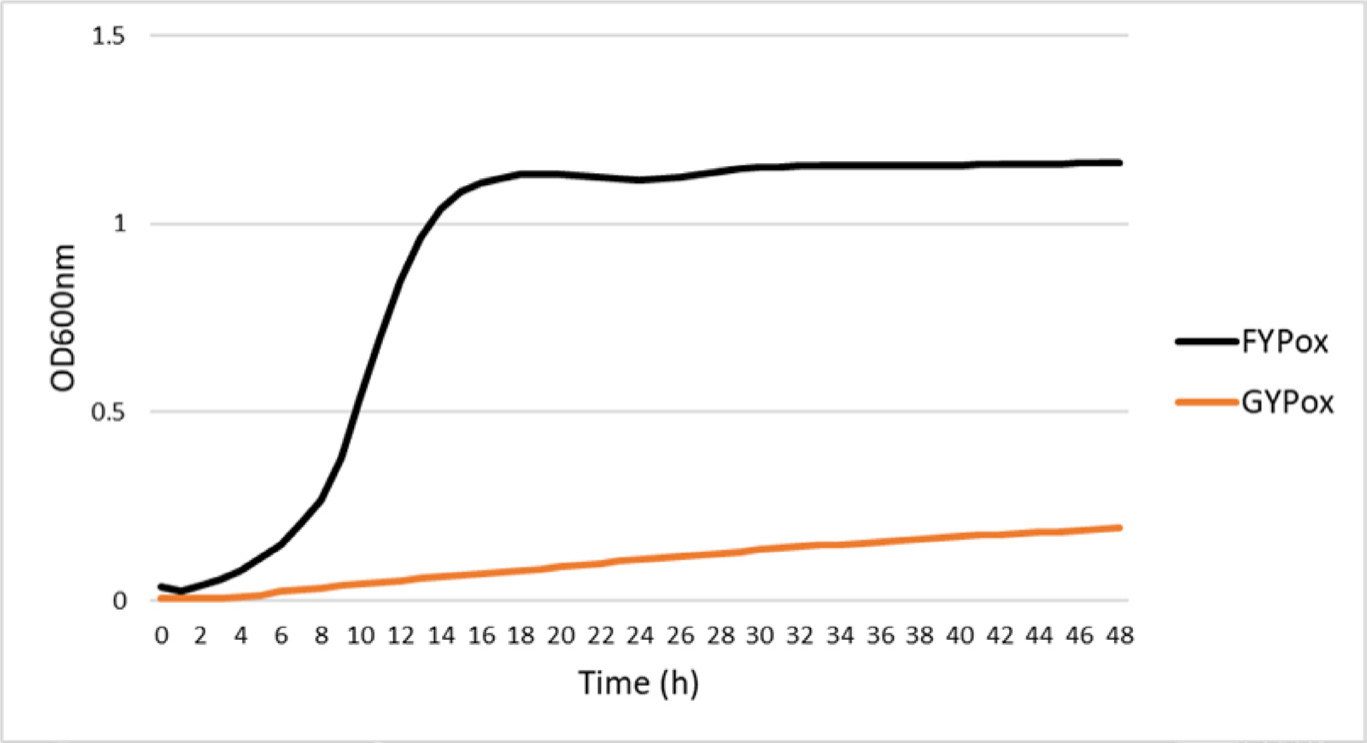
Fructophilic characteristics of Es01^T^. Growth curves of strain Es01^T^ in GYP and FYP under aerobic (ox) conditions on a microtitre plate.

Strain Es01^T^ required the addition of 1% (w/v) d-fructose to the general LAB de Man–Rogosa–Sharpe (MRS) medium for optimal growth, suggesting its fructophilic character. This was confirmed by growth experiments in FYP and GYP media (d-fructose was replaced by d-glucose in the latter medium). In microtitre plates (microaerophilic conditions), a growth rate of 0.142±0.010 h^−1^ was determined for FYP at 30 °C with a final OD_600_ of 1.134±0.081 after 18 h of growth (Fig. 3). However, very poor growth was observed in GYP (growth rate of 0.003±0.001 h^−1^ and final OD_600_ of 0.166±0.015). Strong aeration (5 ml medium in 50 ml flasks and 200 r.p.m.) resulted in enhanced growth in GYP (OD_600_ of 1.408±0.018 in GYP compared to 1.102±0.018 in FYP after 24 h). The OD_600_ at 24 h in GYP under static conditions was 0.022±0.000 and 0.070±0.024 for anaerobic (Anaerogen, Oxoid) and aerobic conditions, respectively. Growth in FYP resulted in OD_600_ of 0.302±0.003 and 0.312±0.012 under similar conditions, respectively, evidencing that the presence of oxygen (strong agitation) enhanced growth also with fructose. Similarly, growth on plates was obtained in FYP, GYP, MRS and MRS supplemented with 1% fructose (MRSF) under aerobic conditions but, in contrast to FYP or MRSF, no or very poor growth was observed in GYP or MRS plates under anaerobiosis (Anaerogen). Growth in MRSF under static conditions (microaerophilic) occurred at pH 4.5, but not at pH 3.5. Growth was also observed at 15 °C in this medium and conditions after more than 120 h of incubation, but not at 42 °C.

**Fig. 4.**
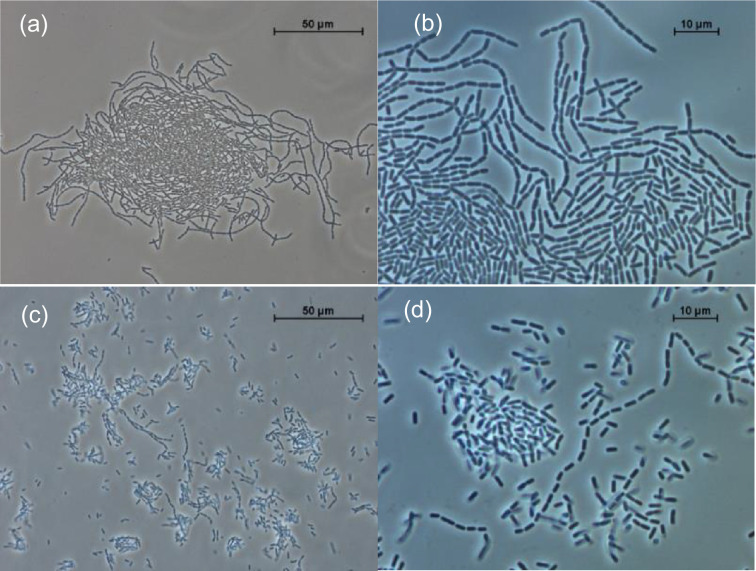
Microscope images of Es01^T^ culture in liquid (×40x (**a**) and ×100 (**b**)) or solid (×40 (**c**) and ×100x (**d**)) media.

The strain was very sensitive to salt concentration and addition of 0.5% NaCl (w/v) inhibited growth. The range of fermentable sugars was limited: only d-glucose, d-fructose, maltose, sucrose and turanose were used out of 49 carbohydrates present in the API50CH gallery. On MRSF agar plates Es01^T^ developed as round, smooth colonies of 1–2 mm diameter with a white appearance. The cells had a rod shape (2.5±0.4×1.1±0.1 µm) and when grown in liquid medium they were generally present in the form of long chains (median of 31 cells per chain), compared to solid medium (median of 4 cells per chain; Fig. 4).

## *Nicoliella*, a fructophilic LAB genus

Genome inspection of *N. spurrieriana* SGEP1_A5^T^ and Es01^T^ showed that these strains lack a gene encoding the bifunctional alcohol/acetaldehyde dehydrogenase (AdhE) which is present in heterofermentative LAB. This characteristic is shared with the genus *Fructobacillus* and some species of *Apilactobacillus* and explains their poor growth on glucose and their preference for fructose [3]. A putative aldehyde dehydrogenase gene was encoded in Es01^T^ (R4146_07225), but it was absent in SGEP1_A5^T^. The fructophilic character of *N. spurrieriana* SGEP1_A5^T^ has already been described [7]. Similar to *N. spurrieriana* SGEP1_A5^T^ and other FLAB [1], the presence of oxygen resulted in an enhancement of growth on glucose for Es01^T^. Glucose growth stimulation by oxygen in SGEP1_A5^T^ was less important compared to Es01^T^. However, differences in culture aeration between the two studies may account for this. As for strain SGEP1_A5^T^, the search for Mdh-encoding genes in the Es01^T^ genome resulted in one gene (locus R4146_02355), with its product showing 59.4% amino acid identity with the characterized Mdh from *Leuconostoc mesenteroides* (accession number CAD31644.1) [31]. The percentages of identity between putative Mdh in *Apilactobacillus* species, a close FLAB relative of *Nicoliella*, and *L. mesenteroides* Mdh were also found to fall within the range of 60%. This observation suggests that these putative Mdh enzymes may correspond to the enzyme responsible for recycling NADH through the formation of mannitol from fructose.

Apart from the existence of a megaplasmid or secondary chromosome in both strains SGEP1_A5^T^ and Es01^T^, an additional characteristic of these members of *Nicoliella* was the presence of megaproteins encoded in their genomes. These proteins were specific for *Nicoliella*, but the similarity between those from SGEP1_A5^T^ and Es01^T^ was low (Tables S2 and S3). The megaproteins from both *Nicoliella* species were characterized by the presence of repetitions very rich in serine and alanine residues and sometimes by the presence of tandem repeats of DUF5776 domain sequences at their carboxy terminal parts, a domain of unknown function and presumed to form a stalk in surface proteins. Examples of this were the products of the Es01^T^ loci R4146_00595, R4146_01085, R4146_01355, R4146_01375, R4146_05150, R4146_05170 and R4146_06960, which encoded proteins of 2891, 6644, 3764, 4481, 7786, 8370 and 11 708 amino acids, respectively, generally carrying predicted signal peptides for secretion, and that were present in both the chromosome and the megaplasmid (7.3% of the whole genome). In addition, other genes encoding proteins sharing the above-mentioned characteristics and with more than 1000 amino acids were also encoded in the Es01^T^ genome. The presence of these giant proteins, although noteworthy, is not extraordinary in prokaryotes [32]. They usually consist of surface proteins which may participate in bacterial fitness by increasing its ability to persist in their niches [32]. In fact, the presence of clusters of genes encoding such giant extracellular proteins (also covering up to 7% of the genome and with products ranging from 3000 to 9000 amino acids) has been reported in multiple strains of the FLAB *Apilactobacillus kunkeei* [33], a species phylogenetically close to *Nicoliella* and which can also be isolated from similar niches (flowers and insects). The two *Nicoliella* isolates that have been obtained so far originate from flowers and from honey, but other partial 16S rRNA sequences derived from unidentified LAB isolates from the gut of bees from the genera *Trigona*, *Heterotrigona*, *Meliponula* or *Melipona* around the world [34,35] and from non-cultured bacteria from corbiculate bees gut [36], display more than 98% identity with the Es01^T^ 16S rRNA gene, indicating a clear host-associated pattern for *Nicoliella* members.

## Proposal of *Nicoliella lavandulae* sp. nov.

Based on the phylogenetic results, as well as low ANI and dDDH values compared to the closest species, *N. spurrieriana*, we conclude that strain Es01^T^ represents a new species, for which the name *Nicoliella lavandulae* sp. nov is proposed. The type strain is Es01^T^ (= CECT 30999^T^=DSMZ 117325^T^=CCM 9394^T^), isolated from flowers of *Lavandula angustifolia*.

## Description of *Nicoliella lavandulae* sp. nov.

*Nicoliella lavandulae* (la.van.du'lae. N.L. fem. gen. n. lavandulae, named after the genus of plants from which it was isolated).

Cells are Gram-positive, catalase-negative, non-motile, short rod-shaped and typically occur in chains of variable length depending on growth conditions. Colonies are 1–2 mm in diameter, round and smooth with a white appearance on MRS supplemented with fructose. Obligately fructophilic, growth with glucose extremely poor under anaerobiosis or low oxygen conditions. Es01^T^ degrades sugars through the phosphoketolase pathway and aerobic conditions enhanced growth with both glucose and fructose. Growth occurs between 15 and 37 °C but not at 42 °C. Es01^T^ grows poorly at pH 5.5 and not at pH 4.5 and addition of 0.5% NaCl completely inhibits growth. Acid is produced from d-glucose, d-fructose, maltose, sucrose and turanose. It cannot ferment glycerol, erythritol, d-arabinose, l-arabinose, d-ribose, d-xylose, l-xylose, d-adonitol, methyl β-d-xylopyranoside, d-galactose, d-mannose, l-sorbose, l-rhamnose, dulcitol, inositol, d-mannitol, d-sorbitol, methyl d-mannopyranoside, methyl α-d-glucopyranoside, *N*-acetyl-glucosamine, amygdalin, arbutin, aesculin, salicin, cellobiose, maltose, lactose, melibiose, trehalose, inulin, melezitose, raffinose, starch, glycogen, xylitol, gentibiose, d-lyxose, d-fucose, l-fucose, d-arabitol, l-arabitol, gluconate, 2-keto-gluconate or 5-keto-gluconate.

The type strain, Es01^T^ (=CECT 30999^T^=DSMZ 117325^T^=CCM 9394^T^), was isolated from *Lavandula angustifolia* flowers. The GenBank accession number of its 16S rRNA gene is OR978307. The Whole Genome Shotgun project of ES01^T^ has been deposited at DDBJ/ENA/GenBank under the accession JAWMWH000000000. The version described in this paper is version JAWMWH010000000.

## supplementary material

10.1099/ijsem.0.006497 Uncited Table S1.

## Abbreviations

ANI: average nucleotide identity
dDDH: digital DNA–DNA hybridization
FLAB: fructophilic lactic acid bacteria
LAB: lactic acid bacteria
MRS: de Man–Rogosa–Sharpe
