# Corrigendum: Nicoliella lavandulae sp. nov., a novel fructophilic Nicoliella species isolated from flowers of Lavandula angustifolia

**DOI:** 10.1099/ijsem.0.006538

**Published:** 2024-09-27

**Authors:** Cristina Alcántara, Ángela Peirotén, Luis A. Ramón-Nuñez, José M. Landete, Manuel Zúñiga, Vicente Monedero

**Affiliations:** 1 Laboratorio de Bacterias Lácticas y Probióticos, Instituto de Agroquímica y Tecnología de Alimentos (IATA-CSIC), Av. Agustín Escardino 7, 46980 Paterna, Spain; 2 Departamento de Tecnología de Alimentos, National Institute for Agricultural and Food Research and Technology (INIA-CSIC), Carretera de La Coruña Km 7.5, 28040 Madrid, Spain; 3 Valencian Institute for Agricultural Research (IVIA), 46113 Valencia, Spain

In the published version of this article, there was an error in the Author Notes section.

The first paragraph should read ‘The GenBank accession number of 16S rRNA gene sequence of strain Es01^T^ (=CECT 30999^T^=DSM 117325^T^=CCM 9394^T^) is OR978307’ instead of ‘The GenBank accession number of 16S rRNA gene sequence of strain He02^T^ (=CECT 31001^T^=DSM 117324^T^=CCM 9395^T^) is OR978307’.

