# Supplementary material for: Nicoliella lavandulae sp. nov., a novel fructophilic Nicoliella species isolated from flowers of Lavandula angustifolia
Source: Int J Syst Evol Microbiol. 2024 Aug 27;74(8):006497. doi: 10.1099/ijsem.0.006497 (PMC11349052; doi:10.1099/ijsem.0.006497)
Supplement: Uncited Table S1. [file ijsem-74-06497-s001.pdf]

Supplementary Table 1. Estimates of evolutionary divergence between Es01 and N. spurrieriana 16S rRNA sequences

	16S_1|3543..5118c	16SRNA2|541019..542594	16S3|655618..657193	16S4|712179..713754	16S5|975215..976790
16S_1|3543..5118c					
16SRNA2|541019..542594	0,0012702013				
16S3|655618..657193	0,0012702013	0,0000000000			
16S4|712179..713754	0,0012702013	0,0000000000	0,0000000000		
16S5|975215..976790	0,0025431372	0,0012702013	0,0012702013	0,0012702013	
CP093361.1:c344199-342624_Nicoliella_spurrieriana_strain_SGEP1_A5_chromosome_complete_genome	0,0115422425	0,0115446981	0,0115446981	0,0115446981	0,0128414299
CP093361.1:1057983-1059558_Nicoliella_spurrieriana_strain_SGEP1_A5_chromosome_complete_genome	0,0115422425	0,0115446981	0,0115446981	0,0115446981	0,0128414299
CP093361.1:c1542852-1541277_Nicoliella_spurrieriana_strain_SGEP1_A5_chromosome_complete_genome	0,0115342190	0,0115366711	0,0115366711	0,0115366711	0,0128324855
CP093361.1:c161451-159876_Nicoliella_spurrieriana_strain_SGEP1_A5_chromosome_complete_genome	0,0128386944	0,0128414299	0,0128414299	0,0128414299	0,0141409972
CP093361.1:c218217-216642_Nicoliella_spurrieriana_strain_SGEP1_A5_chromosome_complete_genome	0,0128386944	0,0128414299	0,0128414299	0,0128414299	0,0141409972

Distances were calculated by Maximum Composite Likelihood [1] including transitions and transversions considering uniform rates among sites and a homogeneous pattern among lineages. Estimation of variance was estimated by bootstrap with 500 replicates.

1. Tamura K., Nei M., and Kumar S. (2004). Prospects for inferring very large phylogenies by using the neighbor-joining method. Proceedings of the National Academy of Sciences (USA) 101:11030-11035.

Supplementary Table 2. Homologies between the megaproteins encoded by the Es01 genome. The coverage (up) and percentage of identity (down) are shown; (–) no blast hit. The blast searches were performed with the proteins encoded by the loci in the “locus tag” column as query.

Locus tag	R4146_00595	R4146_01085	R4146_01355	R4146_01375	R4146_05150	R4146_05170	R4146_06960
R4146_00595		-	6%<br>31.03%	1%<br>55.56%	-	-	-
R4146_01085	-		-	-	-	-	-
R4146_01355	5%<br>31.98%	-		-	-	7%<br>38.60%	7%<br>38.60%
R4146_01375	-	-	14%<br>55.52%		-	5%<br>46.86%	5%<br>46.49%
R4146_05150	-	-	-	-		1%<br>47.32%	11%<br>46.98%
R4146_05170	-	-	-	3%<br>47.41%	6%<br>47.29%		90%<br>94.49%
R4146_06960	-	-	-	-	8%<br>47.84%	63%<br>95.69%	

Supplementary Table 3. Homologies between the megaproteins encoded in the Es01 and *N. spurrieriana* SGEP1_A5^{T} genomes. The coverage (up) and percentage of identity (down) are shown; (–) no blast hit. The blast searches were performed with the Es01 proteins as query (loci R4146_0XXXX).

Locus tag	MOO44_ 04290	MOO44_ 04305	MOO44_ 04310	MOO44_ 04325	MOO44_ 04330	MOO44_ 04335	MOO44_ 04340	MOO44_ 04670	MOO44_ 05160	MOO44_ 07225	MOO44_ 07230
R4146_0 0595	-	-	6%<br>35.14%	-	6%<br>31.82%	7%<br>33.33%	-	-	48%<br>36.00%	6%<br>37.84%	5%<br>35.91%
R4146_0 1085	-	-	-	-	-	-	-	95%<br>44.58%	-	-	-
R4146_0 1355	-	-	-	-	14%<br>42.96%	-	10%<br>43.16%	-	-	7%<br>38.43%	-
R4146_0 1375	-	6%<br>54.33%	11%<br>54.15%	23%<br>57.28%	12%<br>50%	7%<br>62.22%	13%<br>60.56%	-	-	7%<br>44.98%	4%<br>48.54%
R4146_0 5150	-	-	-	-		-	-	-	-	13%<br>48.94%	13%<br>47.76%
R4146_0 5170	0%<br>66.67%	-	3%<br>45.52%	4%<br>29.43%		0%<br>66.67%	4%<br>47.41%	-	-	21%<br>49.70%	15%<br>58.58%
R4146_0 6960	-	-	2%<br>45.52%	-	-	-	3%<br>46.69%	-	-	15%<br>49.70%	6%<br>58.58%
